# Wearable Smartphone-Based Multisensory Feedback System for Torso Posture Correction: Iterative Design and Within-Subjects Study

**DOI:** 10.2196/55455

**Published:** 2025-01-22

**Authors:** Amanda Polin Pereira, Olibario Jose Machado Neto, Valeria Meirelles Carril Elui, Maria da Graca Campos Pimentel

**Affiliations:** 1 Faculty of Medicine of Ribeirão Preto University of São Paulo Ribeirão Preto SP Brazil; 2 State University of Mato Grosso do Sul Nova Andradina MS Brazil; 3 Institute of Mathematics and Computer Sciences University of São Paulo São Carlos SP Brazil

**Keywords:** stroke rehabilitation, posture, postural balance, wearable technology, multisensory feedback, smartphone, stroke, mHealth, mobile health, digital health, digital technology, digital intervention, wearable technology, gerontology

## Abstract

**Background:**

The prevalence of stroke is high in both males and females, and it rises with age. Stroke often leads to sensor and motor issues, such as hemiparesis affecting one side of the body. Poststroke patients require torso stabilization exercises, but maintaining proper posture can be challenging due to their condition.

**Objective:**

Our goal was to develop the Postural SmartVest, an affordable wearable technology that leverages a smartphone's built-in accelerometer to monitor sagittal and frontal plane changes while providing visual, tactile, and auditory feedback to guide patients in achieving their best-at-the-time posture during rehabilitation.

**Methods:**

To design the Postural SmartVest, we conducted brainstorming sessions, therapist interviews, gathered requirements, and developed the first prototype. We used this initial prototype in a feasibility study with individuals without hemiparesis (n=40, average age 28.4). They used the prototype during 1-hour seated sessions. Their feedback led to a second prototype, which we used in a pilot study with a poststroke patient. After adjustments and a kinematic assessment using the Vicon Gait Plug-in system, the third version became the Postural SmartVest. We assessed the Postural SmartVest in a within-subject experiment with poststroke patients (n=40, average age 57.1) and therapists (n=20, average age 31.3) during rehabilitation sessions. Participants engaged in daily activities, including walking and upper limb exercises, without and with app feedback.

**Results:**

The Postural SmartVest comprises a modified off-the-shelf athletic lightweight compression tank top with a transparent pocket designed to hold a smartphone running a customizable Android app securely. This app continuously monitors sagittal and frontal plane changes using the built-in accelerometer sensor, providing multisensory feedback through audio, vibration, and color changes. Patients reported high ratings for weight, comfort, dimensions, effectiveness, ease of use, stability, durability, and ease of adjustment. Therapists noted a positive impact on rehabilitation sessions and expressed their willingness to recommend it. A 2-tailed t-test showed a significant difference (*P*<.001) between the number of the best-at-the-time posture positions patients could maintain in 2 stages, without feedback (mean 13.1, SD 7.12) and with feedback (mean 4.2, SD 3.97), demonstrating the effectiveness of the solution in improving posture awareness.

**Conclusions:**

The Postural SmartVest aids therapists during poststroke rehabilitation sessions and assists patients in improving their posture during these sessions.

## Introduction

The 2019 Global Burden of Disease report indicates that stroke is a major global health issue, with around 12.2 million new cases and 6.6 million deaths worldwide that year. This makes stroke the second leading cause of death and the third leading cause of disability [1], with its occurrence increasing with age and affecting both males and females [2]. In Brazil, stroke is the leading cause of death, and mortality from stroke has increased in recent years. Registering 103,000 deaths in 2019, the number rose to over 112,000 by 2023 [3].

One of the most common and challenging poststroke conditions is hemiparesis, which affects a substantial number of stroke survivors globally [4,5]. For these patients, controlling trunk movement becomes a fundamental motor skill essential for performing various functional tasks [6,7]. For instance, research shows that maintaining proper posture correlates with walking ability in patients undergoing acute stroke rehabilitation [8].

In individuals without hemiparesis, posture typically involves symmetric and balanced alignment of all body parts, including the head, shoulders, spine, hips, and limbs, following the body’s natural curves. In contrast, a person with hemiparesis, characterized by muscle weakness on one side of the body due to brain injuries or stroke, may exhibit an asymmetric posture, resulting in an imbalanced or tilted position. We refer to the term “best-at-the-time posture” to describe the optimal posture achievable by a hemiparetic patient, considering their motor limitations (we use the term “correct posture” interchangeably). The goal is to achieve the highest level of alignment and balance possible.

The literature registers many efforts to support stroke survivors with respect to improving trunk stability [9], trunk compensation [10], motor control [11-13], and accessing mobility [14]. Furthermore, a systematic review has confirmed the significantly positive impact of trunk training on various aspects of trunk control, sitting and standing balance, and mobility [15].

In health care, literature acknowledges the promise of smartphones [14,16] and wearables [17-20] in empowering individuals, aiding diagnosis, promoting behavior change, and enabling self-monitoring. Wearable and rehabilitation devices for different body parts, such as head, limbs, and torso, enhance training outcomes through valuable feedback [21-24]. Moreover, numerous studies have affirmed the feasibility of inertial sensors for balance and gait assessment [25-28], as corroborated by comprehensive literature reviews [29,30], even for individuals with chronic stroke [31].

In the context of wearable technology for poststroke patient support, researchers have delved into various aspects. For instance, studies demonstrated the feasibility of using step activity monitors for patients with recent stroke [32,33]. One study involved the use of intelligent insoles for analyzing the gait of patients with hemiparesis [34]. Another result is a home-based rehabilitation system using wearable sensors in smartwatches, allowing therapists to monitor patients remotely [35]. In another study, the authors evaluated the impact of haptic nudging delivered via a wrist-worn wearable device on upper limb movement during inpatient stroke rehabilitation [36]. Additionally, a recent study reported a test for assessing kinematic parameters in chronic stroke survivors. This test used a standardized mobility assessment with a simple smartphone attached to the lumbar spine using an elastic band to measure participants' kinematics [14].

Existing literature explores a range of wearable and mobile device-based solutions for postural monitoring. In the context of hemiparesis, researchers have investigated the effects of rhythmic haptic cueing on spatial and temporal gait characteristics using haptic feedback [37]. In a broader context, Smart Pose leverages a smartphone’s camera, accelerometer, and magnetometer to detect poor neck posture during smartphone use, providing feedback through vibrations, text messages, and alarms [38]. Additionally, a 3-axis accelerometer biofeedback system corrected neck posture during prolonged computer use and effectively reduced inappropriate neck angles [28]. Other innovations include elastic t-shirts with embedded sensors for posture feedback [39], wearable devices for spine posture monitoring [40], and posture differentiation systems [41].

However, it is essential to recognize that effective functional rehabilitation requires both body awareness and torso control to ensure upper limb functionality [4,7,9,42]. Additionally, concerns regarding the cost and availability of treatment [43-46] highlight the need to explore alternatives that use ubiquitous, low-cost smartphones. Despite the growing number of mHealth mobile apps for patients with stroke [47], few address trunk control as a primary focus [27,48,49]. Furthermore, while both intrinsic and extrinsic feedback are crucial for motor learning after stroke [50-52], and therapists spend a significant amount of time providing this feedback [53,54], few efforts used multisensory feedback from smartphones for upper body rehabilitation [24]. Moreover, while smartphone-based multisensory feedback has proven effective for postural monitoring in healthy individuals [55], similar solutions for patients with stroke are still lacking. This highlights the need for research into new wearable technologies to enhance rehabilitation support and improve posture and trunk awareness in patients with stroke.

To address this challenge, we aimed to develop Postural SmartVest. This affordable wearable technology takes advantage of low-cost smartphone resources by (1) leveraging the built-in accelerometer sensor to monitor sagittal and frontal plane changes continuously and (2) exploiting the device’s visual, tactile, and auditory feedback to guide patients in performing the movements required to return to their best posture at the time.

## Methods

### Overview

The research group comprises 2 occupational therapists (authors APP and VMCE) and 2 computer scientists (authors OJMN and MDGCP). To design the initial software architecture, the group received input from other health and computing professionals [56].

The study was conducted in a specialized rehabilitation center that requires both ethical committee approval and verification of ethical and safety aspects for studies conducted on its premises. Therapists participating had at least 1 year of experience in stroke rehabilitation and received training on device use before working with patients. The smartphones used in the feasibility study were either from the research team, having been tested and shown to function properly, or were participants' own devices. To minimize malfunction risks, no smartphone was allowed to be charged during use by participants. The Informed Consent Form informed participants about potential discomfort due to heat and the vest and assured them that, in the unlikely event of a smartphone malfunction causing an explosion, immediate assistance and emergency services would be provided. Data acquisition and storage were handled securely, with all data kept anonymized and stored in accounts requiring login via a secure network. Photographs were authorized with face identification removed. Participants consented to the use of data for the study and were informed of their right to withdraw at any time without coercion or obligation. Participation was voluntary and did not include any financial benefit, in compliance with Brazilian regulations.

The study comprised design and development iterations involving brainstorming meetings, interviews with therapists and patients, prototype development, feasibility studies, and experimental sessions (see workflow in Textbox 1).

**Research workflow.**

Literature review

*Iteration 1*

- Brainstorm meetings
- Interview with therapists
- Prototype #1
- Feasibility study

*Iteration 2*

- Prototype #2
- Pilot study

*Iteration 3*

- Prototype #3
- Kinematic assessment
- Postural SmartVest

*Within-subjects study*

- Presession interviews with patients
- Experimental session
- Postsession interviews with patients
- Postsession interviews with therapists

Throughout the study, our literature review was ongoing and dynamically aligned with emerging themes. Initially, it concentrated on the specific needs of patients with stroke [4] and the effectiveness of trunk training [15]. Next, we reviewed work on novel wearable [21] and affordable smartphone-based solutions [31], including reports in literature reviews [29,57], and noticed the involvement of healthy adults in feasibility studies [26,58], as well as control participants [27]. These works are representative of the broader ongoing review process, which continued to cover these and additional relevant areas throughout the study.

We used the Google Sheets software for statistical analysis relative to the within-subject study. We used thematic analysis [59] for categorizing interview responses. For the kinematic assessment, we compared angular momentum from values calculated by Vicon and the smartphone’s sensors.

### Participants

Our study involved 3 groups: poststroke participants, therapists, and healthy participants. We used interviews and questionnaires to collect requirements, demographic data, and their impressions of Postural SmartVest.

For the interviews with therapists, the inclusion criterion was having at least 1 year of experience in stroke rehabilitation. We invited professionals from a rehabilitation center using printed flyers and email invitations. In the feasibility study, we recruited participants via university email: the inclusion criteria were being 18 years or older and owning a smartphone. The exclusion criterion was self-reported physical or cognitive impairments. For the pilot and within-subjects studies, the inclusion criteria were having chronic hemiparesis, being 18 years or older, walking independently, having cognitive ability for communication, and attending rehabilitation sessions at least twice a week for a minimum of 1 month.

### Iteration 1: Brainstorming Meetings, Interviews With Therapists, and Feasibility Study

**Figure 1:**
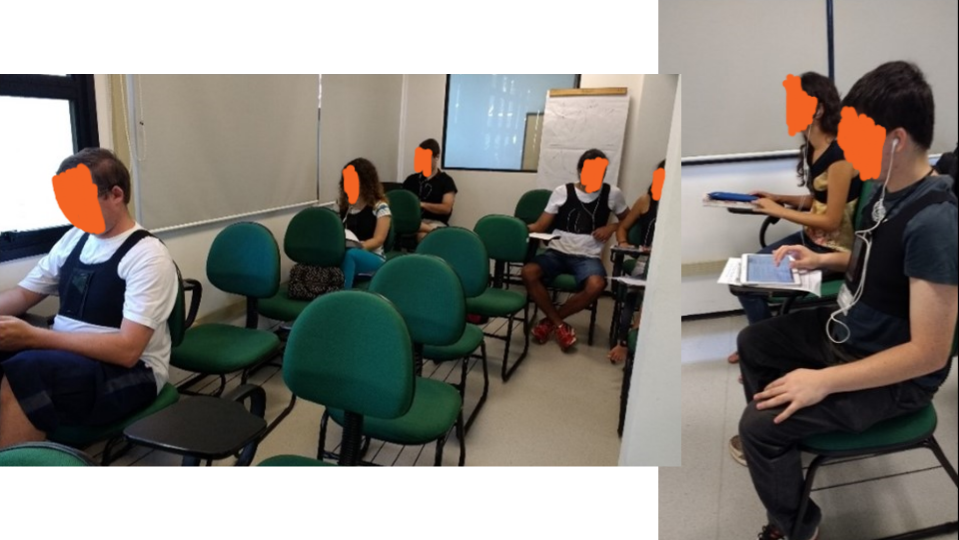
Prototype #1 in use in the deployment phase of the feasibility study.

For the brainstorming meetings, the research team held three 2-hour sessions to gather functional and nonfunctional requirements for the solution to develop the initial prototype comprising the vest and the app, Prototype #1 (Figure 1).

For the interviews with therapists, one team member (APP) conducted semi-structured interviews with therapists. The interview adhered to a structured protocol comprising 8 questions categorized into 4 categories: resources for posture improvement, general technology acceptance, specific technology requirements, and smartphone use (Multimedia Appendix 1: App A).

For the feasibility study, we used the first version of the app. We created 8 vests and conducted sessions with 8 participants at a time. We provided smartphones to participants who could not use their own. Each session lasted 2 hours and followed a 3-phase protocol: preparation, deployment, and follow-up. In the preparation phase, therapists adjusted the vests and calibrated the seating posture for each participant.

In the deployment phase, participants set their devices to airplane mode and wore headphones to ensure privacy for their feedback. They then engaged in smartphone-free activities like reading or using their laptops (Figure 1). The deployment phase consisted of two 30-minute segments: feedback from the app was turned off during the first part and turned on during the second. The app continuously recorded the participants’ postural data throughout both segments in this phase.

In the follow-up phase, participants responded to 2 questionnaires, one with scaled questions and the other with open-ended inquiries. These questionnaires aimed to assess usability and satisfaction. One questionnaire evaluated Postural SmartVest as an assistive technology for posture correction (Multimedia Appendix 1: App B), while the second assessed Postural SmartVest as an assistive technology in general (Multimedia Appendix 1: App C). For the latter questionnaire, we adapted the Quebec User Evaluation of Satisfaction with Assistive Technology (QUEST; version 2.0), a 12-item assessment designed to measure user satisfaction with devices and services [60]. We used a translated and validated version [61] and focused on the device-related section of the questionnaire. This section included 8 scale questions that assessed size, weight, adjustability, safety, durability, ease of use, comfort, and overall effectiveness of the technology.

### Iteration 2: Pilot Study

The feedback from the previous iteration guided the design of Prototype #2. We then used this prototype in a pilot study involving 1 patient with hemiparesis to observe the solution and detail the within-subjects study design. In the pilot study and the following studies, the smartphone used was a Motorola G with Android (version 5.1).

**Figure 2:**
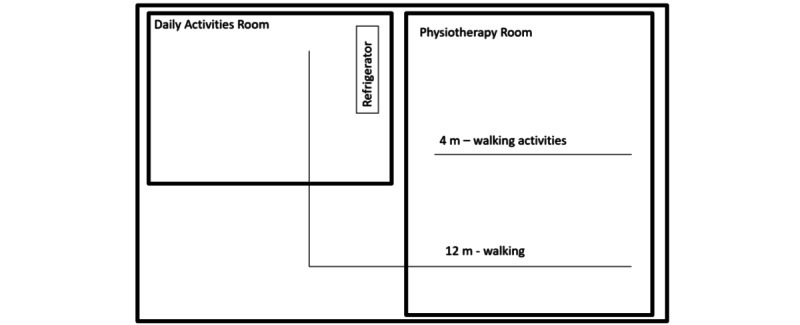
Layout of the therapy room.

During the pilot study, the poststroke patient followed a protocol that included 5 ambulant-based daily activities conducted in the therapy room (Figure 2). The protocol consisted of 4 distinct phases. In the first phase, the participant walked 8 m, incorporating forward, sideways, and backward walking. In the second phase, they proceeded with a 12-m free walk to a refrigerator. In the third phase, the participant engaged in an upper limb activity that entailed opening the refrigerator, taking a glass, and returning it to the fridge. Finally, in the fourth phase, they completed a 12-m free walk back to the starting point.

### Iteration 3: Kinematic Assessment

The feedback from the pilot study informed the development of a new prototype (Prototype #3). At this stage, we checked the accelerometer readings obtained by the app running on Motorola G with Android (version 5.1).

On the one hand, sensor type and placement, activity characteristics, and population-specific conditions can influence the accuracy of sensor outcome estimates, as reported in a study that used research-grade wearable sensors on the upper arms, waist, and ankles and included poststroke patients [62]. On the other hand, the literature reports positive results regarding using state-of-the-art smartphone accelerometer technology. For instance, researchers investigated the comparative performance of 3 different commercially available smartphone accelerometers among themselves and to a gold-standard Vicon MX motion capture system, indicating that the devices are valid and reliable measuring instruments for estimating linear accelerations [63].

We assessed the angle calculations of the third prototype by comparing them with the Vicon Gait Plug-in system, a gold-standard in full-body kinematic and kinetic modeling. The Vicon system includes software and fixed cameras that capture signals from a moving target, providing visual feedback through animated vectors and plans, and it also calculates quantitative data, including distances and angles between selected planes.

**Figure 3:**
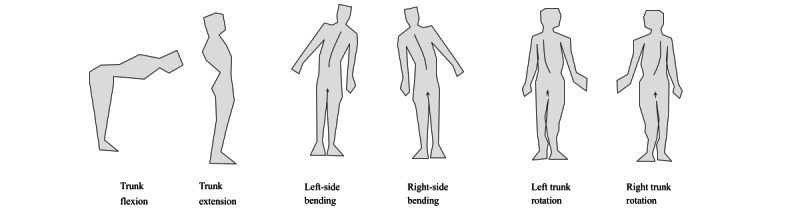
Movements used for simultaneous data collection by Vicon and the app.

Under the guidance of one occupational therapist (APP), author OJMN used the prototype to perform 6 movements: trunk flexion, trunk extension, left and right-side bending, and left and right trunk rotation (Figure 3).

**Figure 4:**
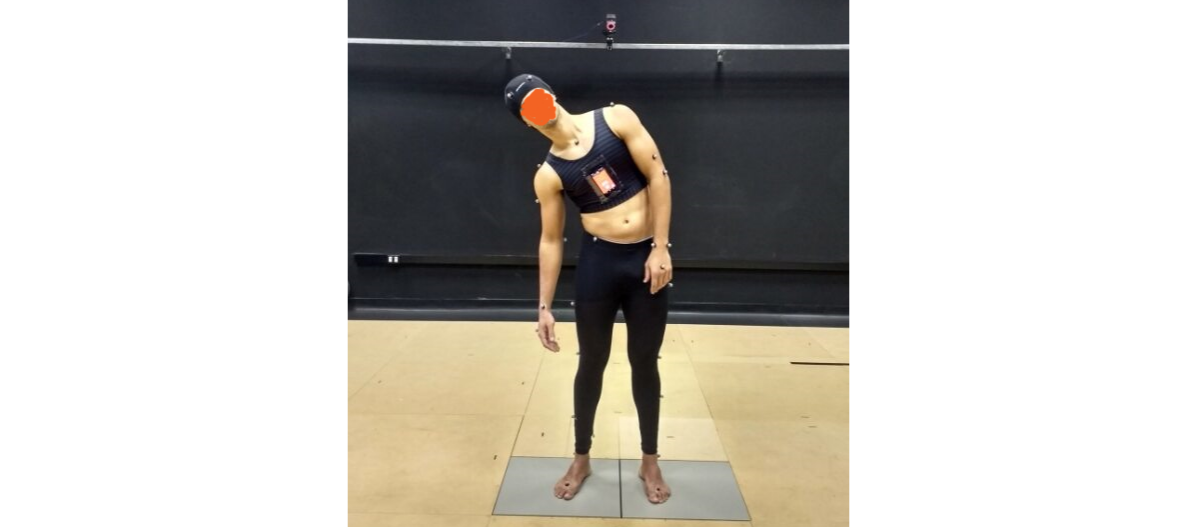
Simultaneous data collection by Vicon and the app.

The therapist calibrated the subject’s optimal posture position, and for all movements, the subject began in an upright position, executed the movement, and returned to the initial position. The Vicon system and the app prototype collected data simultaneously, following an initial manual synchronization process, with the Vicon recording frames at 250 frames per second and the app prototype recording angular moments at each second along with coordinate values. To reconstruct the trunk segment, we placed markers on the clavicle, sternum, cervical vertebra C7, thoracic vertebra T10, and a point located on the medial border of the right scapula. We conducted 6 sessions using 39 markers on a full-body model (Figure 4).

**Figure 5:**
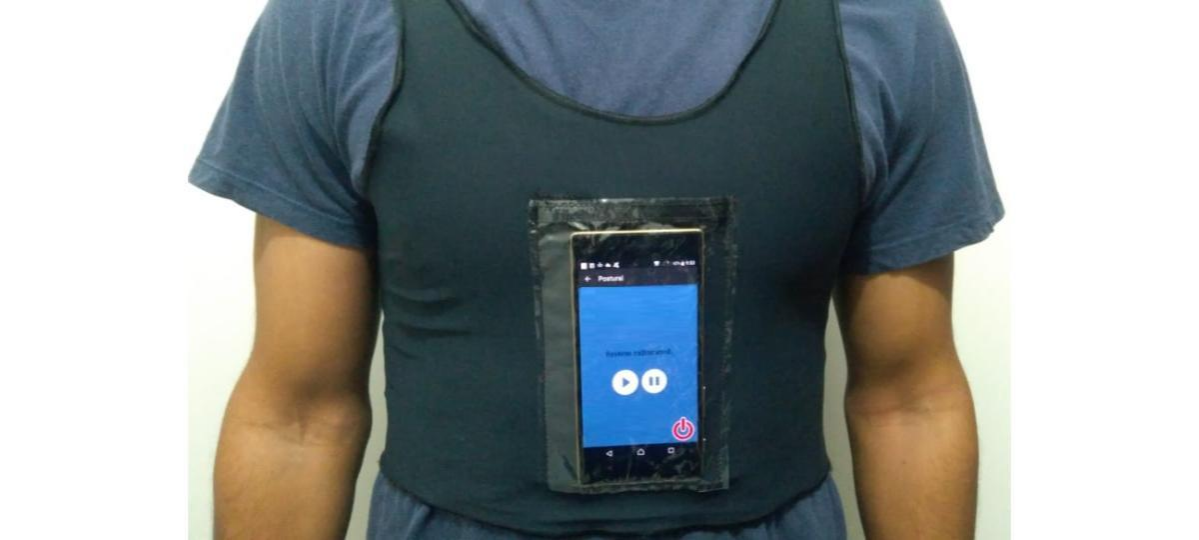
The Postural SmartVest solution comprises a customizable smartphone app placed in a transparent pocket on an athletic, lightweight compression tank top.

Following the kinematic assessment, Prototype #3 was referred to as SmartVest and was used for the remainder of the study (Figure 5).

### Within-Subjects Study

The within-subjects study included individual sessions with poststroke patients, each accompanied by their therapists. Before that, therapists used the Postural SmartVest for about 30 minutes while the researchers provided guidelines to ensure the professionals understood how the solution worked. Only afterward did the therapists apply Postural SmartVest to their patients during their rehabilitation sessions. All sessions used the same smartphone (Motorola G with Android; version 5.1).

In the presession interview with each patient, we gathered information about their posture control during activities, awareness of posture issues, and any potential biases toward one side of the body. We also inquired about their strategies to correct posture problems when they noticed them (Multimedia Appendix 1: App D).

**Figure 6:**
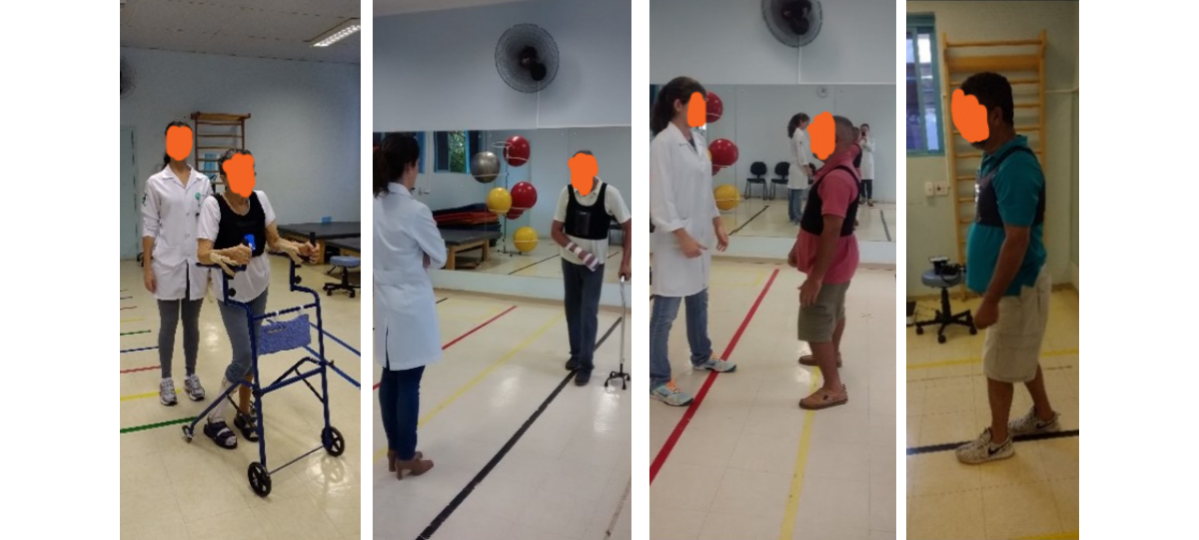
Walking activity.

**Figure 7:**
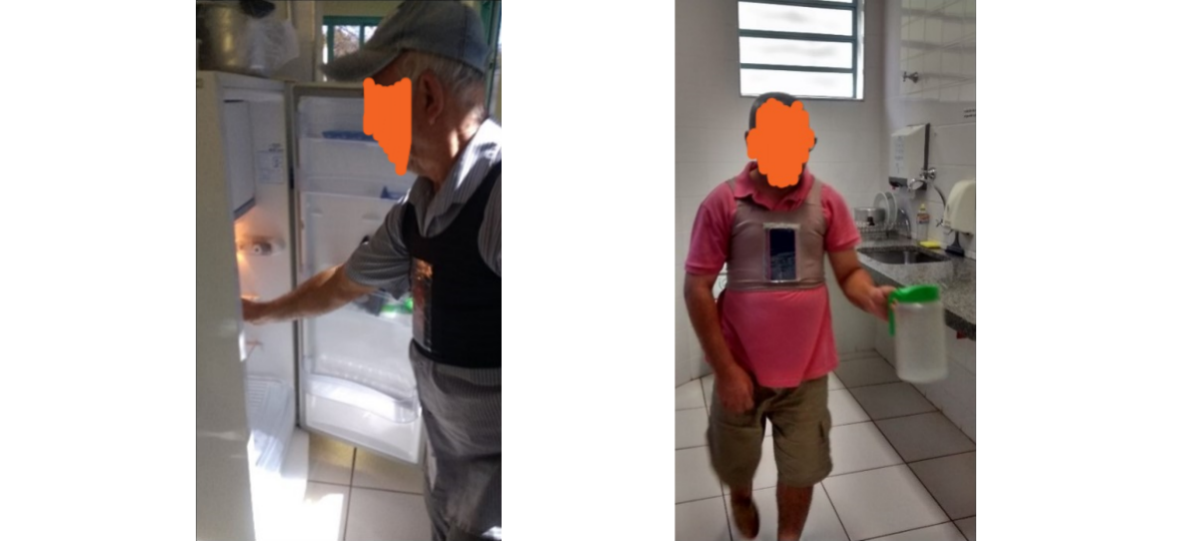
Upper limb activity.

At the beginning of each session, the therapist adjusted the vest on the patient, calibrated the app to recognize the patient’s best-at-the-time standing posture, and initiated the monitoring process (Textbox 2). Subsequently, the patient performed the activities defined in our pilot study twice, in consecutive sessions, each with an estimated duration of 10 minutes (Figures 6 and 7). The app did not provide feedback in the first session but registered all the movements. In the subsequent session, the app provided feedback through screen color changes (green and red), vibrations, and audio guidance.

Afterward, we asked the poststroke patients to fill out 2 questionnaires, one focusing on Postural SmartVest as an assistive technology for posture correction (Multimedia Appendix 1: App B) and the other assessing Postural SmartVest as a general assistive technology (Multimedia Appendix 1: App C).

Additionally, the therapists shared their feedback after working with the patients using a tailored version of the Postural SmartVest QUEST (version 2.0) questionnaire (Multimedia Appendix 1: App E). This study helped us understand the effectiveness of Postural SmartVest during stroke rehabilitation, both from the patient’s and therapist’s perspectives.

**Calibration workflow.**

Adjust the vest on patient

Start the app

Access settings

- Enter the tolerance threshold angle for frontal movement (in degrees)
- Enter the tolerance threshold angle for lateral movement (in degrees)
- Enter the allowable duration for temporary deviations from the calibrated posture (in seconds)
- Ensure the option for vibration feedback is selected
- Ensure the option for audio feedback is selected
- Guide the patient to assume his best posture at that moment
- Press the “Calibrate” button to configure the current position as the best posture for monitoring
- Return to main screen

Press play or pause to initiate monitoring

### Ethics Approval

The study received prior approval from the Hospital of the Clinics of the Ribeirão Preto School of Medicine at the University of São Paulo, Brazil, identifier 57234816.3.0000.5440. As detailed in the text approved by the ethics committee, the study adhered to ethical principles by ensuring beneficence through rigorous monitoring and support for participants, justice by offering equitable access and fair treatment, and respect by obtaining informed consent and addressing potential discomfort and safety concerns proactively.

## Results

### Participants

For the interviews, we enlisted 20 therapists: 10 occupational therapists and 10 physiotherapists. All participants were female, aged 26-40, with varying years since graduation (Table 1).

For the feasibility study, we recruited 28 healthy participants, aged 20-62, with an average age of 30.9. The group included 17 males and 11 females (Table 1). Of the participants, 25 used their own smartphones, while 3 used devices provided by us. The study covered 22 different smartphone models and 7 versions of the Android operating system.

For the pilot and within-subjects studies, we recruited poststroke participants from patients attending rehabilitation sessions at least twice a week for a month at the exact center. Inclusion criteria included chronic hemiparesis from stroke, aged 18 years or older, independent walking ability, cognitive ability for communication, and participation in rehabilitation sessions for at least a month.

We enrolled 1 patient for the pilot study and 40 patients for the within-subjects study (27 males, 67.5%), with a mean age of 57.1 (SD 11.42) years, and functional independence categorized as independent (n=39) of moderate assistance (n=1) on the functional independence measure [64,65]. Among them, 24 had right hemispheric lesions, 16 had left hemispheric lesions, and 38 had ischemic strokes. The duration of their condition ranged from 6 months to 19 years, with a majority between 1 and 4 years (Table 1).

**Table 1:** Participants' demographics.

Characteristics				Values
**Therapists (n=20; all female), n**				
	**Age range (years)**			
		26-30	8	
		31-40	12	
	**Years since graduation^a^**			
		1-2	2	
		3-5	6	
		6-9	9	
		10+	3	
**Healthy participants (n=28; 17 male)**				
	**Age (years)**			
		Range	20-62	
		Mean	30.9	
	**Smartphone used, n**			
		Participant’s own	25	
		Ours	3	
**Poststroke patients in the within-subjects study (n=40; 27 male)**				
	**Age range (years)**			
		20-50	8	
		51-60	16	
		61-70	11	
		71-80	5	
	**Time since stroke (months; mean 43.9 months), n**			
		0-12	16	
		13-24	5	
		25-36	4	
		37-48	5	
		48+	10	
	**Affected hemisphere**			
		Right	24	
		Left	16	
	**Type of stroke**			
		Ischemic	38	
		Hemorrhagic	2	
	**FIM^b^**			
		Moderate assistance	1	
		Moderate or complete independence	39	

^a^Having at least 1 year of experience in stroke rehabilitation.

^b^Functional independence measure.

### Iterative Design

The Postural SmartVest solution comprises a customizable smartphone app for Android devices securely placed in a transparent pocket on an athletic, lightweight compression tank top (Figure 5).

#### Vest Component Evolution

**Figure 8:**
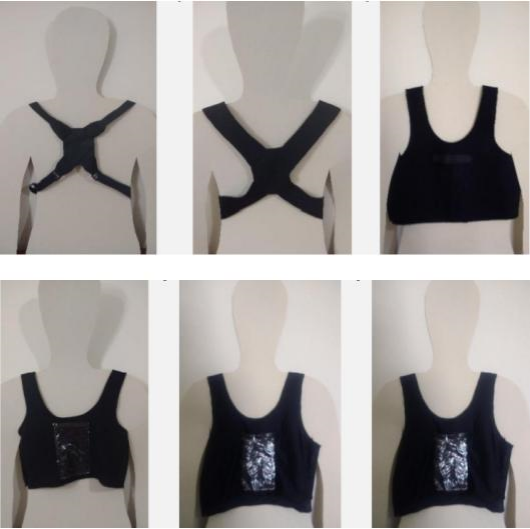
Vest prototypes developed during the iterative design.

The development of the vest component went through several iterations (Figure 8), with 6 prototypes created based on therapist’s input (see responses in Multimedia Appendix 1: App F-G) and feedback gathered during the feasibility study (see responses in the Multimedia Appendix 1: App H). The final version of the garment used in the Postural SmartVest uses an off-the-shelf lightweight compression tank top made from polyamide with elastane. We added a perforated plastic pocket to the top front. The pocket serves 3 primary purposes: securely holding the smartphone, allowing for easy headset connection, and facilitating heat dissipation.

#### App Component Evolution

The smartphone app also underwent significant changes during the iterative design process. Initially conceptualized following brainstorming sessions that defined both functional and nonfunctional requirements (Textbox 3 and Multimedia Appendix 1: App F), the app’s preliminary software architecture was informed by input from health and computing professionals [56].

**Figure 9:**
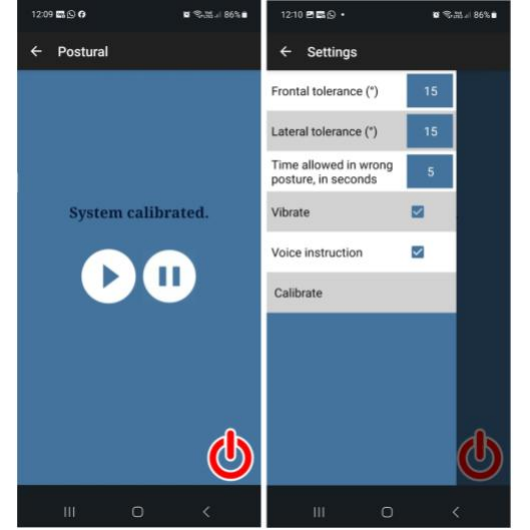
App main screen (left) and configuration panel (right).

Overall, the app was guided by simplicity, feasibility, and adaptability principles, in line with cost-effective assistive technology solutions from our previous research [66]. Refinements and improvements were made based on therapist interviews and feedback obtained during the feasibility study (Textbox 4). The final version has a main screen and a sliding panel for configuration options (Figure 9).

The app records every activity along with its corresponding timestamp. This record encompasses posture changes, feedback delivery, and adjustments to application settings. During the design, we evolved the app to use schemes suggested by the OpenMHealth organization [67] when saving log data.

**App requirements summary.**

*Functional requirements*

- Therapist can calibrate best-at-the-time posture with 1 touch.
- Therapist defines threshold values for small movements.
- Therapist defines threshold values to indicate how long the patient can be off best-at-the-time posture before the app starts guiding the patient.
- The app guides the patient back to their calibrated best-at-the-time posture using screen color, vibration, and audio messages.
- The app must record and store all interactions for later export and analysis.

*Nonfunctional requirements*

- The app should work on a low-end smartphone.
- The smartphone should be attached to a vest within a transparent pocket.
- The app should be compatible with Android, the most common operating system in Brazil.
- The app should work with the screen off to conserve battery if needed.

**App development iterations**

*Prototype #1:*

- Implements all functional and nonfunctional requirements
- Features a single screen displaying x, y, and z coordinates, directions, settings, and a calibration button
- Calibration settings are not stored for future use
- Provides 5 guiding messages in Brazilian Portuguese (1 for each direction and 1 for achieving the best-at-the-time posture)

*Prototype #2:*

- Replaces coordinates and directions with information on sagittal and frontal planes, using values in degrees
- Introduces a new configuration screen for settings and calibration
- Stores and uses calibration settings until a new calibration is performed
- Stores all coordinate readings and feedback provided (version for kinematic assessment)
- Includes play and pause buttons for monitoring, along with an exit button on the home screen
- Expands the number of audio instructions, available in both English and Spanish
- Offers audio feedback every 5 minutes of maintaining the best-at-the-time posture

*Prototype #3:*

- Information on planes renamed to frontal and lateral planes
- Includes a help option with instructions on how to use the app

#### App Component Interface

The app’s main screen provides large buttons for easy access to pausing, resuming, and closing the application (Figure 9, left side). By clicking on "Postural" in the top bar on the left side of Figure 9, therapists can access 6 customization settings, as shown on the figure's right side. From top to bottom in the figure, the first 5 customization parameters allow therapists to configure thresholds for frontal and lateral movements (in degrees), to define the allowable duration for temporary deviations from the calibrated posture (in seconds), and to personalize feedback to the patient, including options for vibration and voice instructions. The therapist uses the last option, Calibrate, to set the best-at-the-time posture that the patient with hemiparesis can achieve at that moment. When calibration is activated, the app registers the current coordinates as the target posture for the patient to achieve and provides audio feedback.

After calibrating, the therapist can return to the main screen using the arrow in the top bar. From that point onward, the therapist can use the play and pause buttons to activate or deactivate posture monitoring. Additionally, we have included a large “off” button to allow the therapist to exit the application easily.

Monitoring starts when the therapist hits the play button, and the whole screen changes to green. If a patient’s posture deviation exceeded the predefined thresholds for lateral and frontal angles relative to the calibrated posture, the app responded immediately with visual feedback, transitioning the screen color from green to red, and tactile feedback through vibrations. Furthermore, the app introduced audio feedback if a patient deviated from the best-at-the-time posture beyond the allowed time for temporary deviations from the calibrated posture. Additionally, the app gives positive feedback every 5 minutes if the patient is maintaining the best-at-the-time posture. This comprehensive feedback system provided patients with real-time guidance tailored to their unique needs and specific postural challenges.

The app provides audio instructions: “please lean forward,” “please lean backward,” “please lean to the right,” and “please lean to the left.” We implemented enhancements throughout the iterations, such as adding positive feedback messages to encourage users to maintain the best-at-the-time posture and random variations in the messages that congratulate users for returning to their best-at-the-time posture.

To meet a requirement identified during the feasibility study, where participants used devices set to various languages, we improved the app to support users in English, Spanish, and Portuguese. It dynamically adapts its interface and voice instructions based on the language settings of the user’s device.

#### Kinematic Assessment

The kinematic assessment results, which involved comparing angular momentum values calculated by the Vicon system with those obtained from the smartphone's sensors, are presented graphically in the Multimedia Appendix 1: App I. The results consistently demonstrated that smartphone accelerometers offer reliable data readings, and our mobile application accurately interprets these values. These outcomes align with existing literature [24,28,38,40,58], reinforcing the utility of our approach within the context of our application. Moreover, this positive evaluation of the system's trustworthiness is in line with the feedback received from therapists regarding its performance and reliability, as detailed in the section *Postsession Interviews With Therapists*.

#### Pilot Study

The pilot study confirmed the proposed activity protocol for the within-subjects study, with participants safely completing the walking circuit within the estimated time frame.

### Within-Subjects Study

#### Presession Interviews With Patients

Findings are summarized in Table 2. Out of the 40 participants with hemiparesis, 34 (85%) acknowledged experiencing challenges in balancing one side of their body. These participants reported various triggers for posture correction, including pain, the sensation of weight, and fatigue. Specifically, 6 participants stated that they only adjusted their posture when they felt pain, 3 did so in response to a sense of heaviness on the side affected by hemiparesis, and 2 did it to alleviate fatigue. Participants mentioned that they recognized the need for correction only when viewing photos (2 participants), standing in front of a mirror (2 participants), or following verbal advice from a family member (6 participants). In total, 4 participants admitted that they often neglected to correct their posture due to forgetfulness, with one of them explaining, “It is hard to remember, and correcting myself all day is tiring.”

When providing opinions on assistive technologies, participants considered the technologies “viable for use” and noted that they provide “progress to believe in improvement.” They also mentioned that these technologies “make rehabilitation more precise” and “help more than just me and the therapist.” Additionally, it was observed that they “stimulate people's lives even with minimal gain.” The repetition of these responses highlights the consistent perception of the technologies' value and potential impact.

Regarding the necessary requirements for the technology, 11 participants agreed that it could assist in the perception and control of the trunk. They envisioned the technology as a “chair that helps maintain correct posture” or “something that allows for posture visualization.” Some participants suggested a device that “corrects the arm and leg to prevent them from falling” and another that “reminds me to correct my posture.” They also proposed features such as “notifying to stay straight” or having the ability to “pull to the side.” Other suggestions included a “vest to correct posture” or a device that “attaches to the trunk and holds it in place.” Additional requirements included providing a “signal on the body,” “holding the shoulders, reminding to keep the back straight, and being fixed on the bra,” and being “close to the body,” “comfortable, practical, and unobtrusive,” and “adaptable to the body.” Participants emphasized that the technology should be “integrated with therapy,” “usable over time,” and “inexpensive.”

**Table 2:** Patients' report on posture, assistive technology, and smartphone usage.

Category		Patients, n
**Maintenance of correct posture**		
	Unable to maintain posture	22
	Able to maintain posture	17
	Able to maintain posture sitting, but not standing	1
**Perception of correct posture**		
	Perceive correct posture	36
	Do not perceive correct posture	4
**Difficulties and postural correction**		
	Remembering to correct and trying to adjust	8
	Unable to correct posture	6
	Need to leave sitting posture to correct	1
**Triggers for posture correction**		
	After perceiving pain	6
	After request from family	6
	After perceiving weight on the side of hemiparesis	2
	After feeling tired	2
	After observing photos	2
	After seeing oneself in the mirror	2
**Opinion on assistive technology**		
	Believe it can help but consider it expensive	13
	Knowledge from TV but no contact	2
	Should be offered by the public health system	1
	Would be good if recommended by professionals	1
**Smartphone usage**		
	Participants with smartphones	26
	Made accessibility adjustments	6
	Experience difficulties	9
	Use daily	19
	Use sporadically	2
	Use only for calls	4
	Use only for WhatsApp, music, and camera	1
	Other uses (WhatsApp, Facebook, YouTube, and internet banking)	12

#### Session Data

The average duration of the stages with and without feedback was 9.14 (SD 5.36) minutes and 8.52 (SD 3.21) minutes, respectively. A 2-tailed *t*-test indicated that these differences were not statistically significant.

We compared the number of the best-at-the-time posture positions patients could maintain in the 2 stages: without feedback and with feedback. The 2-tailed *t*-test results indicated a statistically significant difference between these 2 conditions. Specifically, in the stage with feedback, patients exhibited a higher ability to maintain correct positions (mean 13.1, SD 7.12) compared to the stage without feedback (mean 4.2, SD 3.97). This significant difference (*P*<.001) underscores that patients could achieve and sustain their best-at-the-time postures more frequently when Postural SmartVest provided feedback.

Additionally, we examined the average number of movements performed by patients to attain their best-at-the-time posture in both the stages without feedback and with feedback. In the stage without feedback, patients averaged 8.09 (SD 6.88) movements. In contrast, in the stage with feedback, they averaged 4.49 (SD 1.49) movements. The 2-tailed *t*-test results indicated no statistically significant difference between the values obtained in these 2 stages, with a *P* value of .11.

#### Postsession Interviews With Poststroke Patients

In the evaluation of satisfaction with Postural SmartVest, 37 (92.5%) patients reported being satisfied or very satisfied with the support provided by Postural SmartVest to help them maintain proper posture. In total, 2 respondents had neutral feelings, and 1 expressed dissatisfaction with the solution. In total, 39 (97.5%) patients said they would recommend the solution to others with hemiparesis due to stroke, and one indicated that they might recommend it.

When asked if they were able to maintain their correct posture, 17 patients answered “yes,” 2 responded with “sometimes,” and 21 answered “no.” When asked if they noticed that their body often tilted to a particular side, 18 reported that their body tilted to the right side, 16 indicated that their body tilted to the left, 3 stated that their body tilted backward, and 3 could not respond.

Responses to the questionnaire evaluating Postural SmartVest as an assistive technology using a 1-5 scale (Multimedia Appendix 1: App E) are summarized in Table 3. Regarding open-ended questions directed to the patients, we highlight in Table 4 some critical opinions and the number of users who expressed them.

Poststroke patients provided rich feedback on Postural SmartVest’s usability and effectiveness (Table 4). They suggested design improvements, such as incorporating openings with zippers and using breathable materials for cell phone accommodation. Some recommended extended usage periods, while others emphasized early implementation for gradual posture improvement. Software suggestions included customizable message frequency and varied vibration patterns. Participants highlighted the positive impact of audio feedback, likening it to therapist interactions during sessions. These insights, shared by a significant portion of the 40 patients, showcase the multifaceted utility of Postural SmartVest and its potential to address various poststroke rehabilitation needs. The integration of smartphones into thoracic clothing proved safe during our study, with no observed risks during continuous 1-hour use.

**Table 3:** Patients’ evaluation of postural SmartVest as an assistive technology.

Aspect	Favorable responses, %
Weight	100
Comfort	100
Dimension	97.5
Effectiveness	97.5
Ease of Use	87.5
Stability	82.5
Durability	72.5
Ease of Adjustment	67.5

**Table 4:** Patients’ responses to open-ended questions. The last column indicates the number of participants reporting.

	Responses, n
“I would suggest improvements to the garment, such as an opening Velcro or zipper and the use of breathable material to accommodate the cell phone.”	10
“The vest should be more discreet so that it could be worn more imperceptibly under the shirt.”	10
“The garment should be worn for a longer period, over one hour.”	4
“The solution should be introduced to me as early as the first rehabilitation session, immediately after a stroke, so I could get used to perceiving and correcting my posture earlier.”	4
“I would like to adjust the audio volume of the messages through the app.”	3
“The frequency of the messages should be customizable.”	3
“I would like different vibration patterns for different body movements.”	3
“Listening to the app's directions reminds me of you (the therapist) talking to me during the rehabilitation sessions.”	1
“I would use the solution to prevent falls.”	1
“The solution helps me walk.”	1
“The solution helps me improve my motor perception.”	1
“I believe that the vest alone can play an important role in postural perception.”	1
“I think that using the solution after some rehabilitation sessions is better because right after the stroke, I could not minimally perceive or correct my posture.”	1

#### Postsession Interviews With Therapists

Responses to the adapted QUEST (version 2.0) questionnaire evaluating the solution as an assistive technology (Multimedia Appendix 1: App E) are summarized in Table 5. In total, 2 therapists provided a neutral rating for “Support to Posture” and “Audio guidance accuracy.” They explained that patients with spatial orientation difficulties still required assistance from therapists despite using the solution. In total, 5 therapists provided neutral responses regarding “Solution trustworthiness.” In total, 2 therapists recommended that the app recognize other body parts and suggested improvements in the instructions, noting that the app reinforced the user’s reference sides.

We summarize the responses to evaluating satisfaction with Postural SmartVest on a 1-10 scale (Multimedia Appendix 1: App E) in Table 6. The responses indicated a highly positive impact on patients’ posture control, with 95% rating it as “A lot.” Additionally, 90% of the therapists believed that the app significantly contributed to achieving therapy goals. Moreover, 60% reported conducting fewer patient posture-related interventions after using the app. In terms of ease of use, 70% of the therapists found it “Very easy” for patients to follow the app’s guidance.

**Table 5:** Therapists’ responses to the adapted QUEST (version 2.0) questionnaire (1-5 scale).

Response	Rating 4 or higher, %	Indication
Support to Posture	90	Very satisfied
Audio guidance accuracy	90	Very satisfied
Device comfort	95	Very comfortable
Solution trustworthiness	83	Very reliable
Safety during use	100	Very safe
Likelihood to recommend	100	Very likely

**Table 6:** Therapists’ satisfaction with SmartVest (1-10 scale).

Question	Percentage: answer	Indicating
Did you notice a positive impact on patients’ posture control?	95%: 8 or higher	A lot
Do you think the app contributed to the therapy goals?	90%: 8 or higher	A lot
Did you notice if you conducted more or less posture-related interventions for patients?	60%: 3 or less	Fewer
How difficult was it for the patients to follow the app guidance?	70%: 3 or less	Very easy

## Discussion

### Principal Results

We designed Postural SmartVest, a solution consisting of a chest garment holding a smartphone running an Android application. When the user wears the Postural SmartVest in a rehabilitation session, the therapist first calibrates the app to the best posture the patient can achieve at the time. The app uses the smartphone accelerometer to identify variations in the user’s body movement in the sagittal and frontal planes relative to the calibrated position. Based on these variations, the app provides multisensorial feedback that guides the patient to perform the movements required to return to the calibrated position.

The iterative development of Postural SmartVest, informed by therapist interviews and feasibility study feedback, tailored the final product to user needs. The app reliably identifies posture changes and provides corresponding multisensorial feedback, as substantiated by kinematic assessment.

Postural SmartVest significantly improved patients’ ability to maintain their best-at-the-time postures, with patients showing a higher capacity for correct positions when using feedback. Although the analysis found no significant difference in the number of movements between stages, the increased movement during the feedback stage suggests an active engagement with the system for postural adjustments. This engagement aligns with rehabilitation goals, emphasizing motor skill enhancement and proprioception. These findings highlight Postural SmartVest’s potential as an assistive technology for poststroke patients, emphasizing the importance of feedback in rehabilitation tools.

Postural SmartVest’s flexibility meets each patient’s unique needs and progress, with a highly customizable app enabling therapists to personalize feedback and adjust thresholds. This multisensory approach enhances engagement and may result in more effective posture correction. The app offers a comprehensive feedback system, including visual cues, tactile vibrations, and audio instructions.

The technology received positive feedback regarding its design and usability, with patients and therapists giving favorable ratings for various aspects of Postural SmartVest, including weight, comfort, dimensions, and effectiveness. Patients believed in its utility for walking assistance and motor perception improvement, and their suggestions aligned with their approval. Many patients were willing to recommend Postural SmartVest to other patients with poststroke hemiparesis, highlighting its perceived value. Healthy users also expressed satisfaction with Postural SmartVest’s posture support.

### Limitations

Our study used a specific sample of poststroke patients, which may not fully represent the diverse stroke survivor population. This highlights the need for including a broader and more diverse sample in future research. Although the findings indicate high satisfaction among both patients and therapists, individual preferences and responses varied. Patient feedback identified several limitations that suggest potential improvements to the app's personalization features, such as the ability to adjust the audio volume while using the vest and the inclusion of customizable vibration patterns and prompt frequencies. Additionally, enabling therapists to record guidance prompts in their own voices could enhance the patient experience, as one patient noted: “Listening to the app's directions reminds me of you (the therapist) talking to me during the rehabilitation sessions.”

The study’s duration might have limited the assessment of long-term effects, as improvements in posture maintenance could potentially change over time. A longer follow-up period could provide insights into whether the benefits observed are sustained in the long term. Additionally, the self-controlled approach used in the study—where participants served as their own controls by using the app with feedback turned off—may introduce bias related to user expectations and behavior. Future research should consider alternative control group designs to minimize potential biases and enhance the validity of the results. Another limitation of this study is the relatively unstructured approach to coding and scoring responses from interviews and feedback sessions. The multidimensional nature of the questions, ranging from levels of agreement to broader attitudes and preferences, presented challenges in achieving uniform analysis. To address this, we plan to use more structured, software-assisted procedures in future research to standardize data collection and analysis [68,69], thereby improving the consistency and depth of understanding gained from participant feedback.

Future research should also explore categorizing participants based on injury time, dominance, or body laterality to better understand the varying impacts of the Postural SmartVest. Including studies that involve patients using the device at home could provide insights into its real-world effectiveness and long-term impact in poststroke rehabilitation outside of clinical settings. Understanding how patients integrate the Postural SmartVest into their daily routines and the effects of extended, unsupervised use could reveal valuable information about its benefits in home-based rehabilitation. Furthermore, while Postural SmartVest records a range of activities, including posture changes and feedback delivery, it currently lacks features for therapists to access and use this data effectively. Future research should focus on developing these functionalities to enhance patient monitoring and rehabilitation planning. Additionally, addressing the requirements identified during initial therapist interviews, such as including a photo and video gallery and a dashboard for progress monitoring, could improve Postural SmartVest’s functionality and user satisfaction.

### Comparison With Prior Work

Postural SmartVest is a modified athletic compression tank top with a transparent pocket for a smartphone running a customizable Android app. This app offers tactile vibrations, dynamic visual feedback, and clear audio instructions for posture monitoring and guidance. It is designed to be minimally intrusive, replicable, and useful for both therapists and poststroke patients, using readily available Android smartphones and avoiding the need for expensive specialized equipment.

The development of Postural SmartVest reflects the evolution of cognitive rehabilitation from classical methods to personalized systems with kinematic analysis and user-centered design [70]. It incorporates feedback from patients with stroke and therapists, aligning with recommendations for wearable technology design [71]. Unlike wearable rehabilitation systems that prioritize sensor accuracy over comfort [24], Postural SmartVest is distinguished by its comfort and effective smartphone placement, addressing both functional and user experience aspects.

Research on user perspectives for wearable systems in upper extremity stroke rehabilitation highlights the importance of monitoring both upper extremity and trunk movements and evaluating their quality and quantity [39]. In our experimental sessions, we included activities such as position maintenance, lateral trunk movements, trunk rotation, forward-backward trunk motions, and object retrieval, aligning with tasks assessed by various trunk ability evaluation tools [72]. While some studies have investigated smartphone-based training to improve balance and trunk performance among seated patients with stroke—using modified balance boards with visual and audio feedback in an external monitor [48,49] or smartphones attached to harnesses for screen feedback [25]—Postural SmartVest supports dynamic ambulatory movements and provides visual feedback directly on the smartphone screen, using a single color (red or green) to indicate posture status. This approach is particularly effective in therapy rooms with mirror walls and accommodates individuals with visual deficiencies, which are common after a stroke [73].

Postural SmartVest uses the smartphone’s accelerometer on the patient’s trunk to assist with trunk posture, while many studies use sensors on different body parts for various purposes [74-77]. While postural SmartVest uses smartphone-generated sensor data to identify trunk postures and facilitate posture correction, existing literature often combines data from multiple sensors placed on the user’s body for posture detection [78].

A wealth of research envisions using their solutions to foster collaboration among health professionals [79] or in the patient’s home environment, including studies that use smartphones as a communication component in a remote rehabilitation platform [18,25,33,35,70,76,80,81]. Although we designed Postural SmartVest primarily to assist patients during rehabilitation sessions, we are actively extending our research to the home environment by integrating Postural SmartVest with our experience sampling and programmed intervention platform [68].

### Conclusions

Our goal was to create an affordable wearable, Postural SmartVest, aiding poststroke patients in posture maintenance during rehabilitation. Its simplicity and cost-effectiveness, using widely available devices with efficient sensors, enable multisensory feedback, enhancing accessibility. Patients and therapists expressed utility and satisfaction, emphasizing its clinical potential.

A comparison of patient session data using Postural SmartVest, both with and without feedback, informed essential insights. The significant difference in maintaining correct positions underscored the value of feedback. Although there was no disparity in the number of movements, the increased adjustments during sessions with feedback suggested active patient engagement. These findings emphasize the potential of Postural SmartVest as a valuable assistive technology for poststroke patients and highlight the importance of integrating feedback into rehabilitation tools.

Our study indicates several future research directions, including studying diverse stroke survivor populations, assessing long-term effects, exploring user engagement factors, and evaluating therapist training impact. Additionally, we should prioritize functional outcomes, technology adoption, cost-effectiveness, and addressing unmet needs like enhancing posture visualization and aiding long-term posture monitoring.

## Appendix

Multimedia Appendix 1 Additional material.

## Abbreviations

QUEST: Quebec User Evaluation of Satisfaction with Assistive Technology
