# Supplementary material for: Wearable Smartphone-Based Multisensory Feedback System for Torso Posture Correction: Iterative Design and Within-Subjects Study
Source: JMIR Aging. 2025 Jan 22;8:e55455. doi: 10.2196/55455 (PMC11809616; doi:10.2196/55455)
Supplement: Multimedia Appendix 1 [file aging_v8i1e55455_app1.docx]

**Multimedia Appendix 1**

**App A: Protocol for initial interviews with therapists**

**I. Category: Resources for posture improvement**

1. During the rehabilitation sessions, how do you assist patients in correcting their posture?

2. What resources do you typically use for this purpose?

3. Could you describe the specific activities you engage in to help patients correct their posture?

**II. Category: General technology acceptance**

4. What are your thoughts on the use of technology in the context of health and rehabilitation?

5. Do you believe that technology can be effective in teaching patients to perceive and correct their postures?

**III. Category: Specific technology requirements**

6. From your perspective, what requirements should a new technology designed to help patients perceive and correct their postures meet?

**IV. Category: Smartphone use**

7. Do you own a smartphone?

8. Are you currently using any mobile-based resources to support your rehabilitation sessions?

**App B: Postural SmartVest satisfaction assessment for healthy participants and post-stroke participants**

**I. Satisfaction Assessment (five-point Scale)**

1. Posture support: (Very dissatisfied - Very satisfied)

2. Audio guidance accuracy: (Very dissatisfied - Very satisfied)

3. Device comfort: (Very uncomfortable - Very comfortable)

4. Solution trustworthiness: (Very unreliable - Very reliable)

5. Safety during use: (Very unsafe - Very safe)

6. Likelihood to recommend Postural SmartVest: (Not at all likely - Very likely)

**II. Open-Ended Questions**

7. What is your overall opinion of the application, and do you have any suggestions for improvement?

8. Do you have any additional comments, criticisms, suggestions, or compliments?

**App C: Adapted Quebec User Evaluation of Satisfaction with Assistive Technology (QUEST 2.0) for healthy participants and post-stroke participants**

**I. How satisfied are you with (five-point scale):**

1. The dimensions (size, height, length, width) of your assistive device?

2. The weight of your assistive device?

3. The ease of adjusting (fixing, fastening) the parts of your assistive device?

4. How safe and secure your assistive device is?

5. The durability (endurance, resistance to wear) of your assistive device?

6. How easy it is to use your assistive device?

7. How comfortable your assistive device is?

8. How effective your assistive device is (the degree to which your device meets your needs)?

**App D: Protocol for initial interviews with post-stroke patients**

1. Can you maintain the correct posture during your activities?

2. Are you aware of any instances of incorrect posture? Do you notice if one side of your body tends to exhibit incorrect posture more frequently?

3. How do you adjust your posture when you recognize it as incorrect?

**App E: Postural SmartVest assessment for therapists**

**I. Satisfaction of Postural SmartVest as an assistive technology (adapted from QUEST 2.0) (five-point Scale)**

1. Posture support: (Very dissatisfied - Very satisfied)

2. Audio guidance accuracy: (Very dissatisfied - Very satisfied)

3. Device comfort: (Very uncomfortable - Very comfortable)

4. Solution trustworthiness: (Very unreliable - Very reliable)

5. Safety during use: (Very unsafe - Very safe)

6. Likelihood to recommend Postural SmartVest: (Not at all likely - Very likely)

**II. Impact on patient during treatment (Ten-Point Scale)**

7. Did you notice a positive impact on patients’ posture control? (None - A Lot)

8. Did you notice if you conducted more or fewer posture-related interventions on the patients? (Fewer - More)

9. How difficult was it for the patients to follow the app guidance? (Very Easy - Very Difficult)

10. Do you think the app contributed to the therapy goals? (Not at All - A Lot)

**III. Open-Ended Questions**

11. What is your overall opinion of the application, and do you have any suggestions for improvement?

12. Do you have any additional comments, criticisms, suggestions, or compliments?

13. How did the device affect your therapy session, positively or negatively, and why?

**App F: Requirements for the app**

**F.A Functional Requirements (FR)**

FR 1: The app must allow the therapist to configure the patient’s best-at-the-time posture with the touch of a button.

FR 2: The app should allow the therapist to define threshold values relative to the calibrated posture so that small movements are ignored.

FR 3: The app should allow the therapist to define threshold values to indicate the minimum duration for a posture to be considered incorrect.

FR 4: Upon identifying an incorrect posture, the app should inform the patient by changing the screen color to red and emitting a vibration signal or audio message, or both.

FR 5: After informing the patient of an incorrect posture, the app should emit audio messages guiding the patient of the movements she must perform to return to the calibrated position.

FR 6: Once the patient reaches the calibrated position, the app must change the screen color from red to green and emit an appropriate audio message.

FR 7: The app must store all occurrences during the interaction to be for later analyzed.

**F.B Non-Functional Requirements (NFR)**

NFR 1: The only necessary technology is a low-end smartphone.

NFR 2: The smartphone should be fastened to the patient’s chest using a vest that allows the patient to visualize the device’s screen in the clinic’s mirrors.

NFR 3: The application should be compatible with Android, the most common operating system used in Brazil (according to statcounter.com).

NFR 4: The application should work with the screen turned off to save battery life.

**App G: Therapists' Interview Responses**

**1. During rehabilitation sessions, what do you do to assist patients in correcting their posture?**

Therapists highlighted that they use the following resources for posture improvement during sessions: verbal instructions (100%), direct manipulation (50%), visual and proprioceptive feedback (80%), written instructions (10%), demonstration of incorrect postures, and questions for self-correction (10%).

**2. Which resources do you utilize?**

Therapists indicated that they use mirrors (95%), therapy balls, therapeutic bandages, positioning rollers, illustrations with adhesive tape, their own bodies for demonstration, tactile proprioceptive stimulus (tapping), vests, platforms, chairs, weights, and footrests. These resources are used for various activities, such as walking, sitting, talking, and reaching for objects on shelves or in the fridge.

**3. What is your opinion on technologies for health and rehabilitation purposes? Do you believe that certain technologies could help you teach patients to perceive and correct their postures?**

All therapists agreed that technological resources could assist them in teaching patients to perceive and correct their postures.

**4. What requirements should a new technology for helping patients perceive and correct their postures meet?**

Therapists listed the following requirements for a technology aimed at helping patients perceive and correct their postures: a biofeedback system (mentioned by 11 participants), ease of use (mentioned by 9 participants), affordability, contact with the patient’s skin or trunk, audio, visual, and tactile feedback, small size for mobility, a photo and video gallery to record correct posture, a dashboard with comparative data to monitor patient progress, sample images of correct and incorrect postures, line-based simulations showing postural deviations, and notifications regarding the patient’s postural alignment.

**5. Do you own a smartphone? Do you use any mobile-based resources to support rehabilitation sessions?**

All therapists use apps on their smartphones to support sessions, primarily for functions like a stopwatch, music player, step tracker, and camera.

**App H: Results from the Feasibility Study**

Most of the 28 volunteers used their smartphones during the experiment and sent us the activity log saved on their devices via email or Bluetooth. The study involved devices from 20 different models and six different Android versions. The log data revealed that during the initial thirty minutes, while the app feedback was off, users tended to position their lower back too far from the chair’s backrest, causing their trunks to lean forward. However, in the second part when the app’s feedback was on, postural errors decreased among all users, indicating an increased awareness of maintaining a more appropriate posture.

In their responses to the Postural SmartVest satisfaction questionnaire, at least 55% of participants gave the highest rating to all five aspects evaluated by the questionnaire: maintaining the correct posture, accuracy of the audio guidance, comfort when using the device, trust in the solution, and safety of use. None of the participants gave the lowest score.

In their responses to the questionnaire evaluating the device as an assistive technology, in terms of comfort, two volunteers found the top comfortable but somewhat challenging to put on, while another suggested adding a zipper to the garment for easier wearing. Four participants reported occasional instances where they believed they were in an incorrect posture, but the app did not provide a warning. Three volunteers gave a neutral score for the "maintenance of correct posture" item, and one participant found the "trust in the solution" aspect to be unreliable. Investigation of these cases revealed that the app failed to record data and update sensor values promptly. This was attributed to the presence of numerous open background applications and processes on the devices in question.

**App I: Results from the Kinematic Assessment**

The following figures provide an overview of the kinematic comparison for various trunk movements while data were collected simultaneously by the app and the Vicon system.

The therapist calibrated the subject’s optimal posture position, and for all movements, the subject began in an upright position, executed the movement, and returned to the initial position. Both the Vicon system and the app prototype collected data simultaneously, following an initial manual synchronization process, with the Vicon recording frames at 250 frames/second, and the app prototype recording angular moments at each second along with coordinate values.

Figure 1 displays the simultaneous data capture of left-side bending using both the app (A) and the Vicon system (B). Figure 2 showcases the corresponding data for right-side bending. Figure 3 presents the recorded data for Trunk Extension. Figure 4 exhibits the kinematic analysis results for Trunk Flexion. Figure 5 illustrates the findings regarding Trunk Left Rotation. Figure 6 demonstrates the kinematic analysis outcomes for Trunk Right Rotation.


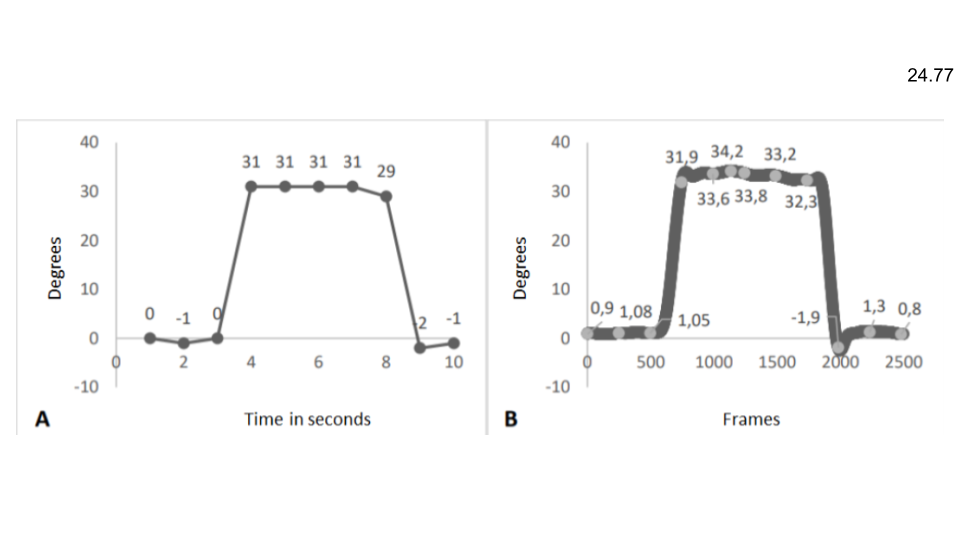


**Figure 1**: left side bending for the app (A) and Vicon(B).


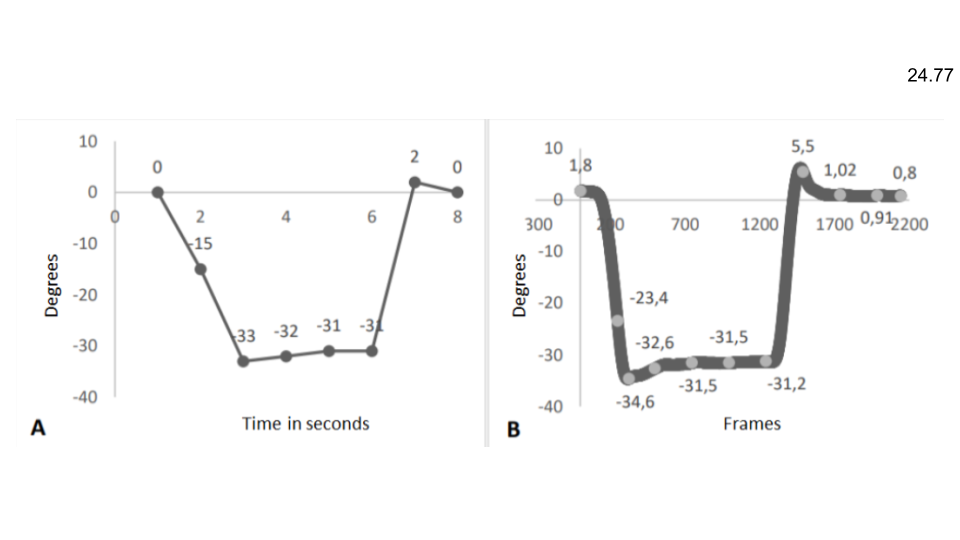


**Figure 2**: right side bending for the app (A) and Vicon (B).


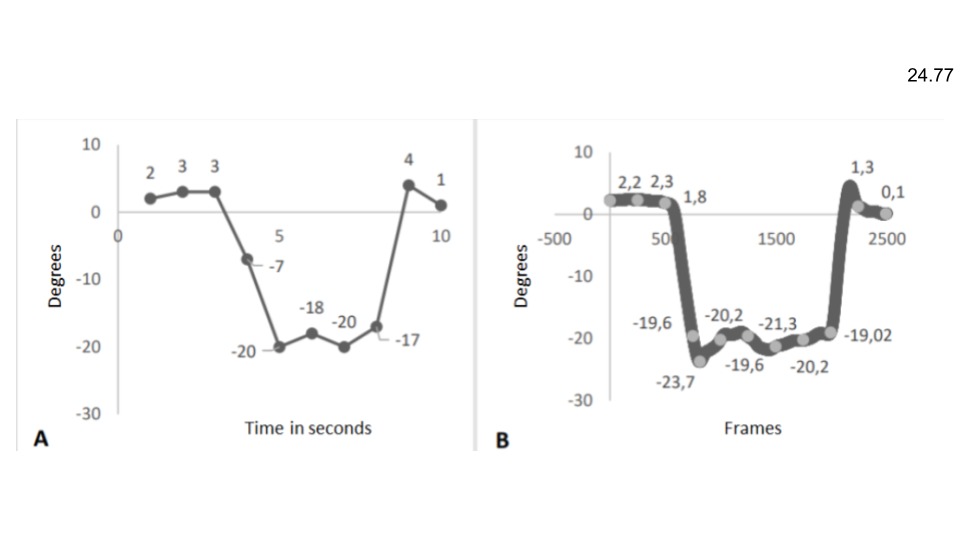


**Figure 3**: trunk extension for the app (A) and Vicon (B).


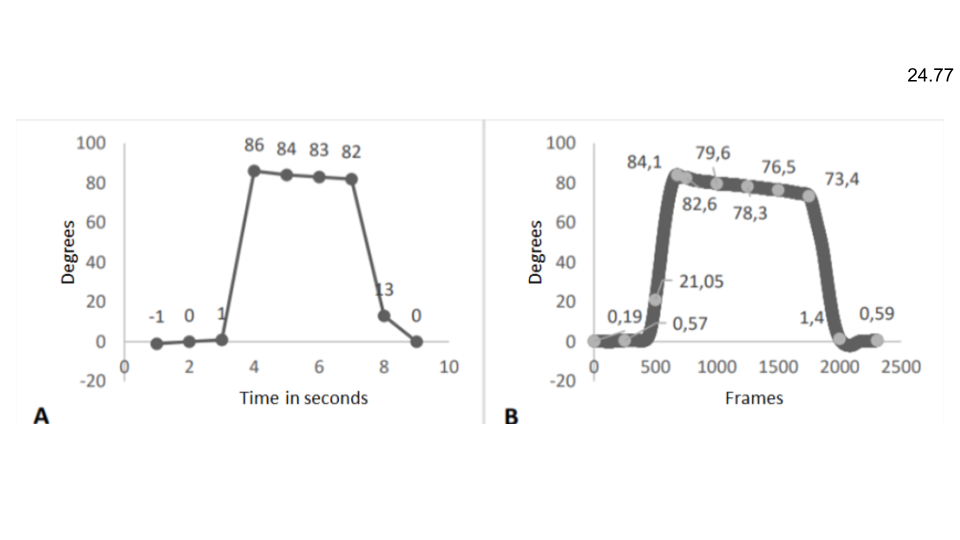


**Figure 4**: trunk flexion for the app (A) and Vicon (B).


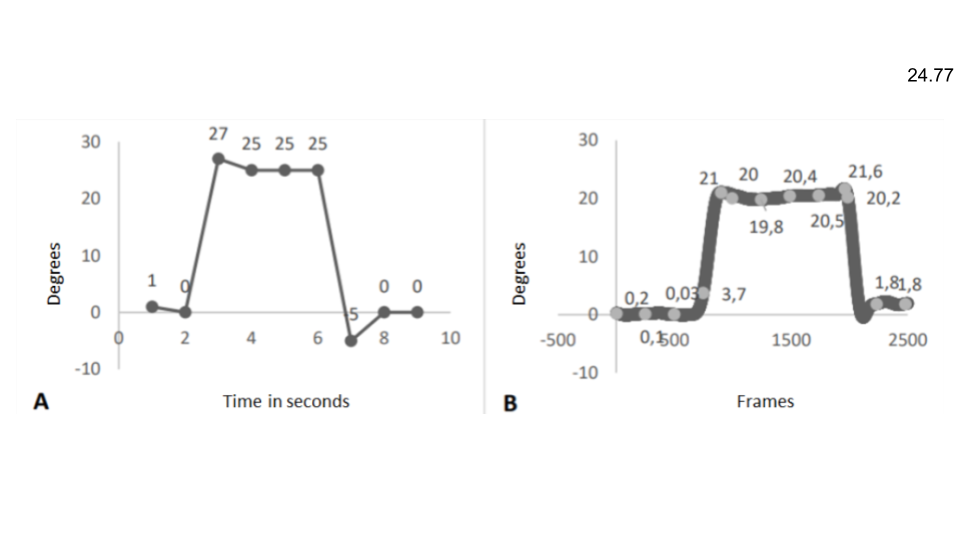


**Figure 5**: trunk left rotation for the app (A) and Vicon(B).


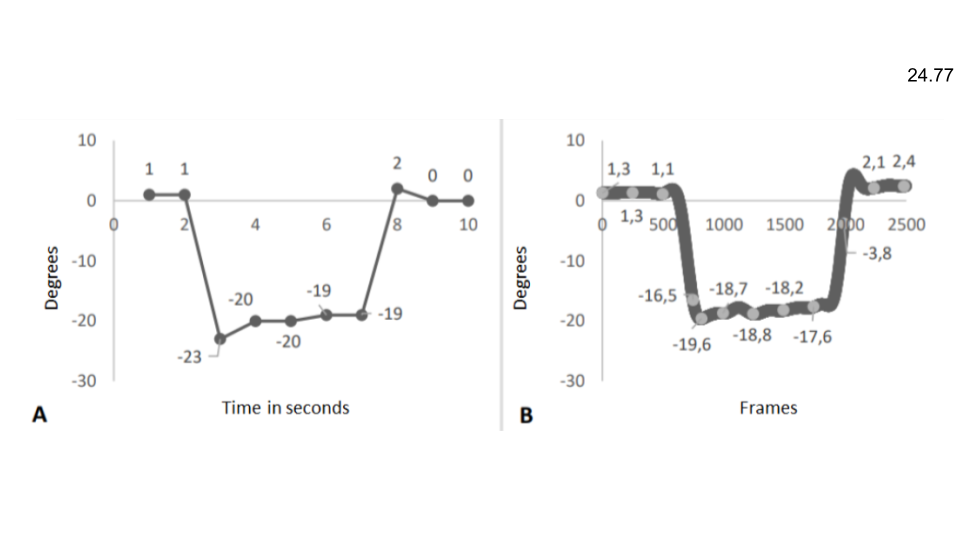


**Figure 6**: trunk right rotation for the app (A) and Vicon (B).
